# Effect of Nutrition Education in NAFLD Patients Undergoing Simultaneous Hyperlipidemia Pharmacotherapy: A Randomized Controlled Trial

**DOI:** 10.3390/nu13124453

**Published:** 2021-12-13

**Authors:** Won Myung Lee, Jea Hurn Bae, Young Chang, Sae Hwan Lee, Ji Eun Moon, Soung Won Jeong, Jae Young Jang, Sang Gyune Kim, Hong Soo Kim, Jeong-Ju Yoo, Young Seok Kim

**Affiliations:** 1 Department of Internal Medicine, Digestive Disease Center and Research Institute, SoonChunHyang University School of Medicine, Bucheon 14584, Korea; 112806@schmc.ac.kr (W.M.L.); mcnulty@schmc.ac.kr (S.G.K.); liverkys@schmc.ac.kr (Y.S.K.); 2 Department of Food Science and Nutrition, SoonChunHyang University Bucheon Hospital, Bucheon 14584, Korea; jhbae@schmc.ac.kr; 3 Department of Internal Medicine, Division of Gastroenterology and Hepatology, SoonChunHyang University School of Medicine, Seoul 04401, Korea; chyoung86@gmail.com (Y.C.); jeongsw@schmc.ac.kr (S.W.J.); jyjang@schmc.ac.kr (J.Y.J.); 4 Department of Internal Medicine, Division of Gastroenterology and Hepatology, SoonChunHyang University School of Medicine, Cheonan 31151, Korea; stevesh@sch.ac.kr (S.H.L.); khskhs@sch.ac.kr (H.S.K.); 5 Clinical Trial Center, Department of Biostatistics, SoonChunHyang University Bucheon Hospital, Bucheon 14584, Korea; moon6188@schmc.ac.kr

**Keywords:** non-alcoholic fatty liver disease, hyperlipidemia, health education, nutrition, nutrition education

## Abstract

Background: Patients with non-alcoholic fatty liver disease (NAFLD) have a high prevalence of combined hyperlipidemia. The importance of nutritional education is well-known in NAFLD, but the impact of medical nutrition therapy (MNT) is unclear in patients with NAFLD with hyperlipidemia. The purpose of this study is to investigate the effect of MNT on the improvement of steatohepatitis in patients with NAFLD taking antihyperlipidemic medications. Methods: Nondiabetic patients with dyslipidemia were prospectively randomized (1:1) either to the MNT group or the control group with standard advice for 48 weeks with simultaneous statin/ezetimibe combination pharmacotherapy at three tertiary centers in Korea. Results: Sixty-six patients were enrolled. Among them, 18 patients dropped out and, overall, 48 patients (MNT group 27, control group 21) were prospectively analyzed in the study. The serum ALT level at 48 weeks between the two groups was not significantly different (66.6 ± 37.7 IU/L vs. 57.4 ± 36.7 IU/L, *p* = 0.40). Serum liver enzymes, controlled attenuation parameter and fibrosis-4 index were significantly improved within the MNT group after 48 weeks compared to baseline. There was no significant difference between the two groups other than the NAFLD fibrosis score (*p* = 0.017). Conclusions: Although there were no significant differences between the two groups in terms of steatosis, metabolic and fibrosis surrogate indicators after 48 weeks, MNT groups showed significant improvement within patient analysis over time. Future studies with a larger number of subjects and a longer study period regarding the effect of MNT are warranted.

## 1. Introduction

Non-alcoholic fatty liver disease (NAFLD) is the most prevalent cause of chronic liver disease worldwide, and is predicted to be the mainstay of liver-related mortality in the foreseeable future [1]. In Korea, the prevalence of NAFLD is 31.0% to 32.8%, and the incidence of NAFLD is 45.1 per 1000 person-years in meta-analysis [2,3].

Hepatic dysfunctions related to hepatic fat accumulation is associated with other metabolic abnormalities, such as type 2 diabetes mellitus, cardiovascular disease and dyslipidemia [4,5], thus leading to increased all-cause mortality and especially cardiovascular mortality [6,7,8]. While NAFLD is perceived as the hepatic manifestation of metabolic syndrome (MetS), the relationship between components of metabolic syndrome is bi-directional and interactive, making it complex to fully comprehend its mechanisms [9]. The heterogenous nature of the disease accounts for the lack of approved pharmacotherapy for NAFLD, and therefore current management strategies of NAFLD focus on lifestyle modifications (e.g., weight loss, healthy diet and increased physical activity) and the mitigation of comorbidities associated with metabolic syndrome [10,11,12].

Dyslipidemia is considered as one of the most common comorbidities in NAFLD patients and should be treated to normalize serum lipid levels. Additionally, dyslipidemia is defined as the presence of one or more abnormal serum lipid concentrations, and it is the main risk factor related to cardiovascular disease. In these patients, antihyperlipidemic agents are a crucial component along with lifestyle modification in the treatment of dyslipidemia. The benefits and safety of antihyperlipidemic agents in NAFLD patients with dyslipidemia were established by previous studies [13]. However, its beneficiary effects were evaluated mostly from a cardiovascular perspective rather than the improvement of hepatic steatosis or fibrosis itself.

The importance of a healthy diet as a part of lifestyle modification in NAFLD patients with or without dyslipidemia is of great interest. However, defining “healthy diet” is a challenging process. Cultural and regional differences in diet intake further add difficulty in applying guideline-supported diets such as the Mediterranean diet or the hypocaloric diet [14]. Even so, the role of nutritional management and its effect is further established in other chronic diseases, notably in chronic kidney disease (CKD) and T2DM. Both CKD and T2DM guidelines suggest that evidence-based medical nutritional therapy (MNT) is of substantial importance [15,16]. MNT in T2DM and CKD is accepted as medical practice and is covering by insurance in Korea. Similarly, consistent nutritional education and dietary recommendation may contribute to improved clinical outcomes in NAFLD patients with dyslipidemia when combined with pharmacologic treatment. Although previous studies have attempted to evaluate the effect of MNT in NAFLD patients, the long-term effects of MNT remain unknown because most of the studies had relatively short duration within 3 to 6 months [17,18].

The purpose of the study is to evaluate the effect of 48 weeks of intensive nutritional education on the improvement of steatohepatitis in patients with NAFLD taking antihyperlipidemic medications.

## 2. Materials and Methods

### 2.1. Patients and Study Design

This was a multicenter, prospective, randomized clinical trial at two tertiary referral centers performed from May 2018 to March 2020. The inclusion criteria were: (1) patients of ages between 19 and 75 who were clinically suggestive of NAFLD with evidence of NAFLD in radiographic studies (ultrasonography, magnetic resonance imaging or computed tomography) or documented steatosis by liver biopsy, (2) serum aspartate aminotransferase (AST) or alanine aminotransferase (ALT) > 40 IU/L and (3) patients with dyslipidemia requiring antihyperlipidemic pharmacologic treatment according to Korean dyslipidemia guideline [19]. Exclusion criteria were: (1) the use of hepatotoxic drugs or drugs known to cause hepatic steatosis, (2) a known liver disease other than NAFLD, including viral hepatitis (detected with positive serum hepatitis B surface antigen or hepatitis C viral RNA), (3) glycated hemoglobin (HbA1C) > 6.5% or currently receiving diabetes medication, (4) serum triglyceride > 500 mg/dL, (5) clinical or biochemical evidence of decompensated liver disease (serum total bilirubin > 3 mg/dL, albumin < 2.8 g/dL, presence of jaundice or ascites), (6) a history of hepatic encephalopathy or variceal bleeding within 6 months prior to study enrollment, (7) glomerular filtration rate < 60 mL/min/1.73 m^2^ and (8) those who refused to participate in this study. Written informed consent for study enrollment was obtained from all patients. This study was approved by the Institutional Review Board of each hospital (SCHBC 2017-11-011-003). This trial was registered in the Clinical Research Information Service (CRIS), which is a member of WHO International Clinical Trials Registry Platform (registration number KCT0002890, date of registration 23-May-2018). Reporting of the study conforms to the Consolidated Standards of Reporting Trials (CONSORT) 2010 statement [20].

### 2.2. Randomization and Concealment

Patients were randomized in a 1:1 ratio either into the nutritional education group or the control group. The randomization of the group was managed by a statistician who was not participating in the study process by using a randomized program of Excel. The randomization and allocation processes were blinded to the investigators and patients. After randomization, antihyperlipidemic agent (Rovazet Tab^®^ HK Inno. N company, Seoul, Korea; rosuvastatin 10 mg + ezetimibe 5 mg, combination drug) to treat dyslipidemia and nutritional education in each group was started. The MNT group was provided with antihyperlipidemic medication plus intensive nutritional education specific for NAFLD by a professional nutritionist with more than 10 years of experience. By contrast, the control group was provided with antihyperlipidemic medication and simple nutritional education paper materials during their regular visit to the outpatient clinic. Both groups were treated and followed up for 48 weeks. Variables were evaluated every 24 weeks until 48 weeks.

### 2.3. Evaluation of Patients

At baseline, all patients underwent clinical and anthropometric (e.g., height, weight and body fat percentage) evaluation and a detailed interview to obtain social habits. Clinical and anthropometric measures were followed up during their routine follow up visits. Laboratory tests including liver function tests, routine biochemistry, lipid profiles and diabetes-related tests (fasting glucose, insulin, HbA1C) were performed every 12 weeks. Obtained laboratory values were used to calculate noninvasive metrics such as homeostasis model assessment technique-insulin resistance (HOMA-IR), the controlled attenuation parameter (CAP) score, the NAFLD fibrosis score (NFS), the hepatic steatosis index (HSI), and bioelectric impedance analysis (Inbody 970^®^, Inbody Inc, Seoul, Korea).

All patients underwent ultrasonography and transient elastography (FibroScan^®^ 502 touch, Echosens, Paris, France) performed by three experienced operators (KYS, KSG or YJJ) blinded to clinical data at baseline and after 48 weeks. The entire clinical trial process and collected investigation items are presented in Supplementary Table S1.

### 2.4. Diagnosis of Fatty Liver

In this study, fatty liver was defined by using radiographic studies (ultrasonography, magnetic resonance imaging or computed tomography) or liver histology. In USG examination, the severity of fatty liver was graded as normal, mild (grade I), moderate (grade II), or severe (grade III) according to the echogenicity of the liver parenchyma; grade I: increased hepatic echogenicity, but periportal and diaphragmatic echogenicity is still appreciable; grade II: increased hepatic echogenicity obscuring periportal echogenicity; and grade III: increased hepatic echogenicity obscuring periportal echogenicity, as well as that of the diaphragm [21].

### 2.5. Protocol of NAFLD Specific Nutrition Education (MNT Group) and Nutritional Education Paper Materials (Control Group)

At baseline, all participants were subjected to a nutrition quotient for adults and the 24 h recall method to evaluate baseline dietary methods [22]. The nutrition quotient investigated balance, diversity and moderation. Dietary methods were analyzed with Can-Pro 5.0, a nutritional evaluation program of the Korean Nutrition Society, to evaluate the daily intake of nutrients, the intake rate compared to nutritional intake standards, and the composition ratio of caloric nutrients. Based on the nutrition quotient and dietary method analysis, the MNT group received face-to-face nutritional education regarding the adequacy of current dietary habits, calorie restriction and ideal macronutrient composition by two professional nutritionists with more than 10 years of experience. Face-to-face nutritional education was conducted 5 times—at 0 weeks, 12 weeks, 24 weeks, 36 weeks, and 48 weeks. Additionally, once a month, phone monitoring was conducted to evaluate the level of dietary practice and provide feedback. At each visit, the degree of achievement of the planned nutrition plan goal and dietary habits was evaluated, and participants were encouraged to comply with the feedback. The MNT groups were also asked to keep a food diary, and the nutritionists gave feedback to the patients based on the diary.

The control group was provided with a brochure on dietary information for fatty liver patients. The brochure provided information on recommended daily calorie intake, recommended food types and examples, and why dietary adjustment is inevitable in patients with NAFLD. The contents of the original brochure are provided in the Supplementary Materials (Figure S1).

### 2.6. Primary and Secondary Outcomes

The primary outcome of this study was the difference in serum ALT level between two groups at 48 weeks. Secondary outcomes included differences in hepatic fibrosis surrogate indicators (liver stiffness measured by transient elastography, NAFLD fibrosis score, FIB-4 index), hepatic steatosis factors (ultrasound steatosis grade, CAP score measured by transient elastography), and other metabolic factors (body mass index (BMI), serum AST, low density lipoprotein (LDL), fasting glucose, HbA1C and HOMA-IR) at 24 and 48 weeks.

### 2.7. Sample Size Calculation

The number of subjects in each group was calculated based on the following hypothesis. According to a study published in 2010 [23], among patients diagnosed with NASH by liver histology, the ALT level decreased from 84 to 41 IU/L after 48 weeks of active lifestyle modification. On the other hand, in the control group, the decrease in ALT was insignificant from 85 to 69 IU/L. The resulting estimated sample size was 25 patients per group, or a total subject population of 50 patients, with an alpha value of 0.05 and a power of 90 percent. Considering a 20% drop out rate, 32 patients per group, or a total of 64 patients, were required.

### 2.8. Statistical Analysis

Demographic and clinical characteristics are presented as mean (range) or median (interquartile range (IQR)) for continuous variables and frequency (percentage) for categorical variables. To compare the variables between the nutritional education group and the control group, the independent samples *t* test or the Wilcoxon–Mann–Whitney U test was used for continuous variables and the χ^2^ test or Fisher’s exact test was used for categorical variables. To analyze the change in variables within the same group, the paired *t* test was performed if necessary. All statistical analyses were performed using R version 4.1.2 (The R Foundation for Statistical Computing, Vienna, Austria) or SPSS version 23.0 (IBM Corp., Armonk, NY, USA).

## 3. Results

### 3.1. Baseline Characteristics

**Figure 1:**
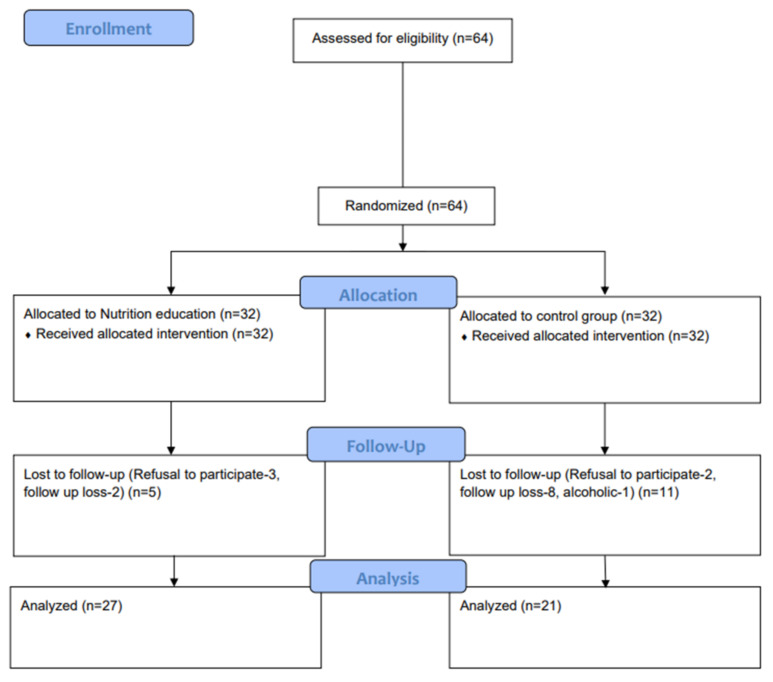
Flowchart showing the flow of participants through the trial.

Sixty-four consecutive patients eligible for the inclusion and exclusion criteria were enrolled and randomized as outlined in Figure 1. Out of these patients, sixteen patients were excluded during the study period because of refusal to participate (*n* = 5), being lost to follow up (*n* = 10) and reported alcoholism (*n* = 1). Finally, a total of 48 patients (27 patients in MNT group and 21 patients assigned in control group) who completed the study process were analyzed. The patients were 41.46 ± 12.41 years old, and 62.5% were males.

Between the MNT group and the control group, no statistically significant difference was observed in any demographic, anthropometric or biochemical parameters. Baseline serum level of ALT was above normal range (73.88 ± 42.55 IU/L). Additionally, the mean serum lipid profile revealed dyslipidemia (LDL-C > 160 mg/dL) adequate for pharmacologic dyslipidemia treatment. The mean values of diabetes-related laboratory values (HbA1C, fasting serum glucose) were in the normal range, excluding T2DM. BMI was above the normal range (>30 kg/m^2^). The values of noninvasive scoring system scores (NAFLD fat score, hepatic steatosis index) and USG fatty liver grade were suggestive of hepatic steatosis. Selected variables are presented in Table 1.

### 3.2. Comparison of Effects of Nutrition Education between Variables

After the 48-week study period, variables were classified into three categories to analyze the effect of MNT from different perspectives; hepatic steatosis, hepatic fibrosis surrogate indicators and metabolic factors. The steatosis factor consisted of the liver function test, the CAP score, the NAFLD fat score, the HSI and the ultrasonographic fatty liver grade. Lipid profiles, anthropometric values and laboratory test for diabetes were classified as metabolic factors. Surrogate indicators representing hepatic fibrosis such as the NAFLD fibrosis score and fibrosis-4 (FIB-4) score were categorized as fibrosis factors. The details of variables and categorization are presented in Table 2.

### 3.3. Effect of Nutritional Education on Serum ALT Level

The serum ALT level between the two groups did not show a significant difference after 48 weeks (*p* = 0.40) (Table 3). However, the ALT level was improved at 48 weeks from baseline within the MNT group (75.81 ± 48.41 IU/L to 57.41 ± 36.66 IU/L, *p* = 0.01), while the ALT level in control group (71.38 ± 34.6 IU/L to 66.57 ± 37.70 IU/L, *p* = 0.051) did not show a significant difference (Table 2).

### 3.4. Effect of Nutritional Education on Steatosis, Metabolic and Fibrosis Factors

**Figure 2:**
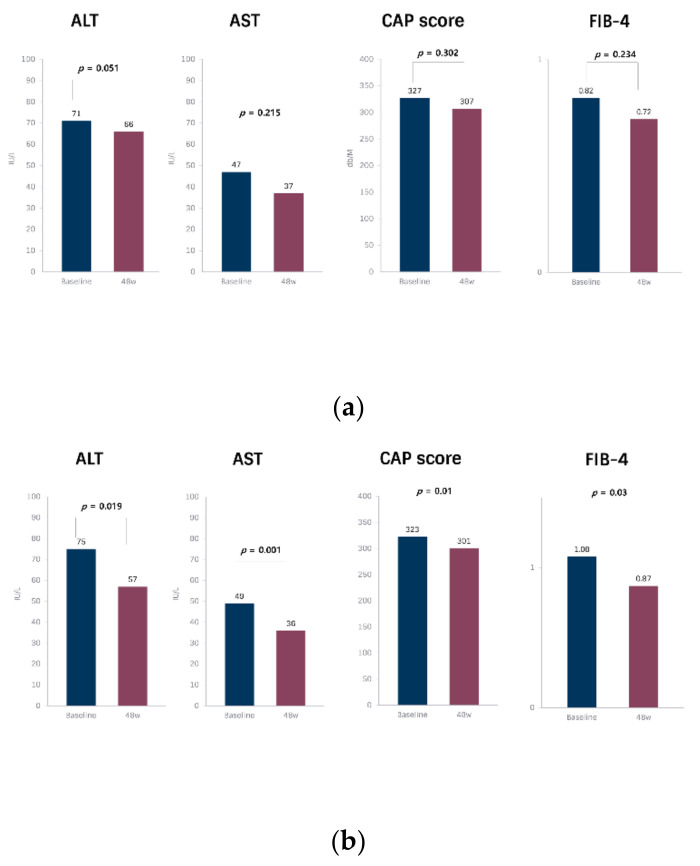
Within patient paired analysis in variables at 48 weeks from baseline within each group. (**a**) Control group; (**b**) MNT group.

When analyzed within each group, AST (*p* = 0.001), CAP score (*p* = 0.01) and FIB-4 (*p* = 0.003) were improved with statistical significance after 48 weeks of nutritional education in MNT group (Figure 2). Among metabolic factors, total cholesterol and LDL cholesterol were significantly improved from baseline in both groups. Fibrosis factors except for FIB-4 were not changed significantly.

Between two groups, there were no significant differences in steatosis, metabolic and fibrosis surrogate indicators when compared both at 24 and 48 weeks, except in the NAFLD fibrosis score (*p* = 0.017) (Table 3).

## 4. Discussion

The etiology of NAFLD involves a complex interplay among various factors, in contrast to single-etiology diseases such as viral hepatitis [24]. Furthermore, NAFLD can be considered either as a cause or a consequence, regarding its relation to metabolic syndrome [25]. Hence, its management strategy requires a multi-disciplinary approach ranging from pharmacotherapy to lifestyle modification, which includes changes in physical activity and diet pattern. However, the current lifestyle modification approach for NAFLD patients, especially from a nutritional perspective, is somewhat ambiguous compared to other chronic conditions, as mentioned above.

Reducing total energy intake is crucial in the diet of fatty liver patients. Three randomized controlled studies demonstrated that reduced energy intake improved weight loss, decreased liver fat mass, decreased liver enzyme levels, and improved insulin resistance [26,27,28]. However, since the appropriate energy intake must be individualized according to the subject’s gender, age, weight, and activity level, collaboration with a food nutritionist is essential.

While the ideal macronutrient composition of diet for NAFLD patients is not established, the importance of a healthy diet in NAFLD and MetS is widely acknowledged. In particular, carbohydrate restriction and the Mediterranean diet are the two most recognized options to be effective for NAFLD patients [29]. This regimen contributes to the reduction in not only NAFLD but also many metabolic contexts such as cardiovascular diseases, hypertension, type 2 diabetes mellitus, obesity, and even cancer [30]

The Mediterranean diet is based on rich monounsaturated fats, plant-derived foods and fruit with limited carbohydrate, red meat and dairy. In particular, the effect of the Mediterranean diet has been widely studied and has been to be proven beneficial in regard to metabolic diseases, NAFLD, morbidity and overall mortality by several previous studies [30,31,32]. However, applying the Mediterranean diet to non-Western populations is a challenging process due to cultural differences in various regions [33]. The traditional Korean diet pattern is composed of salted vegetables, seafood, low saturated fat food, and cereals. However, adaptations to Western lifestyles and diet patterns have increased the prevalence of conditions related to MetS in the past decades.

In this study, we aimed to evaluate the effects of Korean-specific MNT on nondiabetic NAFLD patients with dyslipidemia and its treatment. There was no significant difference between the MNT and control groups after 48 weeks of education in serum ALT. Similar to our study, existing research on specific nutrients and dietary habits have failed to improve steatosis, fibrosis or inflammation. A few reasons may account for this negative result. First, the study participants might have been motivated to comply with the provided nutrition education at the beginning of the study, but their adherence to education contents may have declined over time. As noted on Table 3, certain values (CAP score, total cholesterol, LDL cholesterol, body fat percentage and HOMA-IR) in the MNT group showed favorable results, which were more profound at 24 weeks relative to 48 weeks while without statistical significance. This finding may suggest that continuous adherence to MNT throughout the study period was difficult. Second, the effect of the diet and nutrition education may differ depending on the genetic predisposition such as PNPLA3 or TM6SF2 mutation, so it is necessary to customize it for each individual. However, the same protocol was applied for all the participants in our study [34]. Finally, our study group comprised a relatively small sample size after dropout. This may have affected the study results.

There were no significant differences in steatosis, metabolic and fibrosis surrogate indicators between the two groups after 48 weeks. Nevertheless, serum AST, ALT, CAP score and FIB-4 index showed significant improvements in the MNT group after 48 weeks compared to baseline. A possible explanation for this discordance may include increased attention to the disease and interest in dietary modification by the participants. Additionally, regular visits to the clinic along with MNT may have served as a reminder, encouraging the participants to comply with the recommended diet patterns. These improvements in some variables provide a rationale supporting the potential benefits and necessity of MNT in NAFLD patients.

The strength of this study is in its randomized, controlled, prospective design, along with its aim to establish NAFLD-specific, localized MNT. Despite the attempts of several previous studies to address the effect of MNT in NAFLD patients, randomized controlled trials are scarce and the majority of the reports consist of single-arm, observational studies [35]. Moreover, our study was conducted in Korea, which is a non-Western area. While most of the previous nutrition studies tend to be based on Western countries, the modification of MNT based on regional culture should be considered, since applying the Mediterranean diet without regard for regional difference is not an optimal approach. Additionally, the antihyperlipidemic agent used in this study was statin combined with ezetimibe. Previous studies of antihyperlipidemic agents in NAFLD patients mainly focus on the effect of statin. Existing studies showed relatively little interest in ezetimibe or combined agents.

We acknowledge some limitations in this study. First, a major limitation of our study is that all participants had dyslipidemia requiring pharmacologic treatment. Due to the high variation among subjects in liver pathology and disease stage, the effects of a 48-week lifestyle intervention were difficult to analyze in this relatively small cohort. In addition, MNT superimposed on a pharmaceutical intervention affected liver enzyme results, which also contributed to such difficulties. Hence, the simultaneous initiation of a relatively weak intervention with a relatively strong (pharmaceutical) intervention is a major limitation of this study. Second, the total number of study participants was 48 patients, which is not a large sample size. In a study of this size, it may be difficult to discover the effects of lifestyle change due to the heterogenous nature of NAFLD. Additionally, the overall results may vary even with small changes in patient outcomes. Third, adherence to MNT depended on self-reports by participants in the form of a questionnaire and diary. Such tools are difficult to quantify and rely heavily on patient recall. Finally, the evaluation of steatosis or fibrosis was performed by batteries of non-invasive modalities instead of liver biopsy, which is the most reliable diagnostic tool for hepatic steatosis or fibrosis.

In conclusion, 48 weeks of NAFLD-specific MNT did not show a significant difference when compared to the control group. However, improvements in some variables of the MNT group suggest the potential benefits of MNT. Further studies regarding an appropriate diet therapy with long-term adherence and providing evidence of histological improvement are necessary.

## Figures and Tables

**Table 1:** Baseline characteristics of patients.

Characteristics	All (*n* = 48)	Control (*n* = 21)	MNT (*n* = 27)	*p*
**Demographics**				
Age (years)—mean ± SD	41 ± 12	39 ± 11	42 ± 12	0.390
Sex (male)—number (percent)	30 (62.5)	12 (57)	18 (66.6)	
**Laboratory values**—mean ± SD				
White blood cell count (10^3^/µL)	7.1 ± 2.0	7.7 ± 1.9	6.6 ± 2.0	0.059
Hemoglobin (g/dL)	15.0 ± 1.1	14.9 ± 1.2	15.1 ± 1.0	0.594
Platelet count (10^3^/µL)	260 ± 45	280 ± 49	245 ± 37	0.009
AST (IU/L)	48 ± 28	47 ± 35	49 ± 23	0.891
ALT (IU/L)	73 ± 42	71 ± 34	75 ± 48	0.713
Total bilirubin (mg/dL)	0.8 ± 0.3	0.8 ± 0.4	0.7 ± 0.2	0.355
Serum albumin (g/dL)	4.7 ± 0.3	4.72 ± 0.32	4.78 ± 0.31	0.494
Prothromin time (INR)	1.0 ± 0.1	1.0 ± 0.1	1.0 ± 0.1	0.438
Serum creatinine (mg/dL)	1.0 ± 0.2	1.0 ± 0.2	1.0 ± 0.2	0.688
Serum ferritin (ng/mL)	361 ± 546	436 ± 742	279 ± 149	0.351
**Lipid profiles**—mean ± SD				
Total cholesterol (mg/dL)	239 ± 34	243 ± 33	235 ± 34	0.437
LDL cholesterol (mg/dL)	168 ± 23	171 ± 24	167 ± 22	0.562
HDL cholesterol (mg/dL)	45 ± 9	45 ± 11	45 ± 9.1	0.815
Triglyceride (mg/dL)	188 ± 98	163 ± 62	207 ± 117	0.100
**Diabetes related laboratory values**—mean ± SD				
HbA1C (%)	5.7 ± 0.3	5.7 ± 0.3	5.7 ± 0.3	0.599
Fasting glucose (mg/dL)	106 ± 12	105 ± 10	106 ± 13	0.673
Serum Insulin (uIU/mL)	37 ± 62	28 ± 21	43 ± 81	0.361
C-peptide (ng/mL)	5.2 ± 3.8	4.7 ± 2.5	5.6 ± 4.6	0.370
HOMA-IR	165 ±257	130 ± 92	192 ± 333	0.362
**Anthropometric values**—mean ± SD				
BMI (kg/m^2^)	30.1 ± 4.3	30.0 ± 3.5	30.1 ± 4.8	0.933
Muscle mass (kg)	31.2 ± 8.4	29.4 ± 6.3	32.6 ± 9.6	0.168
Body fat percentage (%)	34.6 ± 8.2	35.3 ± 8.4	34.0 ± 8.2	0.602
**Noninvasive scoring system scores**—mean ± SD				
CAP score (dB/m)	325 ± 56	327 ± 76	323 ± 36	0.840
Fibrosis score (kPa)	7.0 ± 4.2	6.5 ± 2.5	7.4 ± 5.2	0.422
FIB-4	1.0 ± 0.6	0.8 ± 0.4	1.1 ± 0.6	0.106
Hepatic steatosis index	43.6 ± 6.0	43.7 ± 4.9	43.5 ± 6.9	0.909
NAFLD fat score	5.6 ± 9.4	4.3 ± 3.4	6.7 ± 12.3	0.352
NAFLD fibrosis score	−3.1 ± 1.3	−3.5 ± 1.2	−2.8 ± 1.3	0.063
USG fatty liver grade (%)				0.013
Grade I	8 (16.6)	6 (28.5)	2 (7.41)	
Grade II	20 (41.6)	4 (19)	16 (59.2)	
Grade III	20 (41.6)	11 (52.3)	9 (33.3)	

Data were reported as median and interquartile range (IQR) presented as median (25th percentile, 75th percentile) or means and standard deviation (SD) (mean ± SD) for continuous variables. Data were reported as frequency (percentage) for categorical variables. Proportions are presented as percentages for categorical variables. *p*-values were calculated by the Kruskal–Wallis test for continuous variables and chi-square test or Fisher’s exact test for categorical variables. Abbreviations: SD, standard deviation; AST, aspartate aminotransferase; ALT, alanine aminotransferase; INR, international normalized ratio; LDL, low density lipoprotein; HDL, high density lipoprotein; HbA1C, glycated hemoglobin; HOMA-IR, homeostasis model assessment technique-insulin resistance; BMI, body mass index; CAP score, controlled attenuation parameter score; FIB-4, fibrosis -4; NAFLD, non-alcoholic fatty liver disease; USG, ultrasonography.

**Table 2:** Paired analysis in variables at 48 weeks compared to baseline within each group.

Characteristics	Control (*n* = 21)				MNT (*n* = 27)			
	Baseline	48 w	*Mean Difference (95% CI)*	*p*	Baseline	48 w	*Mean Difference* *(95% CI)*	*p*
**Primary outcome**								
ALT (IU/L)	71 ± 34	66 ± 37	−4.8 (−26.8, 17.2)	0.051	75 ± 48	57 ± 36	−18.4 (−33.5, −3.2)	0.019
**Steatosis factors**								
AST (IU/L)	47 ± 35	37 ± 17	−10.3 (−27.1, 0.5)	0.215	49 ± 23	36 ± 15	−12.9 (−20.3, −5.46)	0.001
CAP score (dB/m)	327 ± 76	307 ± 43	−20.2 (−60.2, 19.6)	0.302	323 ± 36	301 ± 44	−22.5 (−39.3, −5.8)	0.010
NAFLD fat score	4.3 ± 3.38	2.9 ± 3.26	−1.3 (−3.2, 0.5)	0.159	6.6 ± 12.2	2.4 ± 1.8	−4.2 (−8.7, 0.2)	0.065
Hepatic steatosis index	43.6 ± 4.8	44.5 ± 4.9	0.8 (−1.3, 3.04)	0.428	43.5 ± 6.8	43 ± 6.1	−0.4 (−2.2, 1.2)	0.565
USG fatty liver grade (%)								
Grade 0	−	−			−	1 (3.7)		
Grade I	6 (28.5)	6 (28.5)			2 (7.41)	11 (40.7)		
Grade II	4 (19)	10 (47.6)			16 (59.2)	7 (25.9)		
Grade III	11 (52.3)	5 (23.8)			9 (33.3)	8 (29.6)		
**Metabolic factors**								
Total cholesterol (mg/dL)	243 ± 33	163 ± 48	−79.8 (−103, −56)	<0.001	235 ± 34	152 ± 42	−83.4 (−103, −63)	<0.001
LDL cholesterol (mg/dL)	171 ± 24	95 ± 36	−75.2 (−93.7, −56.7)	<0.001	167 ± 22	88 ± 37	−78.1 (−95.9, −60.4)	<0.001
HDL cholesterol (mg/dL)	45 ± 11	47 ± 9	1.7 (−1.9, 5.5)	0.336	45 ± 9	48 ± 9	3.3 (0.7, 5.8)	0.012
Triglyceride (mg/dL)	163 ± 62	166 ± 106	2.4 (−38, 2.9)	0.901	207 ± 117	145 ± 51	−62.2 (−107.8, −16.5)	0.009
BMI (kg/m^2^)	29.9 ± 3.5	29.8 ± 3.7	−0.1 (−0.6, 0.2)	0.450	30.0 ± 4.8	29.7 ± 4.5	−0.3 (−0.8, 0.2)	0.251
Muscle mass (kg)	29.3 ± 6.2	29.5 ± 6.3	0.1 (−0.1, 0.4)	0.332	32.5 ± 9.6	31.1 ± 7.4	−1.4 (−3.5, 0.6)	0.164
Body fat percentage (%)	35.3 ± 8.3	36 ± 7.2	0.7 (−2.2, 3.6)	0.620	34 ± 8.2	33.4 ± 8.3	−0.6 (−1.7, 0.4)	0.236
HOMA-IR	130 ± 92	115 ± 99	−15.1 (−70.1, 39.9)	0.573	192 ± 333	100 ± 56	−91.5(−212, 29.3)	0.131
Fasting glucose (mg/dL)	105 ± 10	105 ± 13	−0.1 (−5.3, 5.1)	0.970	106 ± 13	108 ± 11	1.4 (−2.1, 5)	0.402
**Fibrosis factors**								
NAFLD fibrosis score	−3.4 ± 1.1	−3.7 ± 1.5	−0.3 (−0.8, 0.1)	0.186	−2.7 ± 1.3	−2.7 ± 1.3	0 (−0.3, 0.3)	0.998
Fibrosis score (kPa)	6.4 ± 2.5	5.5 ± 1.8	−0.9 (−1.9, 0.1)	0.085	7.3 ± 5.1	5.4 ± 1.4	−1.9 (−3.9, 0)	0.053
FIB-4	0.8 ± 0.4	0.7 ± 0.4	−0.1 (−0.2, 0)	0.234	1.0 ± 0.6	0.8 ± 0.5	−0.2 (−0.3, 0)	0.003

Data are presented as mean ± standard deviation in continuous variable or number (percent) in categorial variables. Proportions are presented as percentages for categorical variables. *p*-values were calculated by the Kruskal–Wallis test for continuous variables and the chi-square test or Fisher’s exact test for categorical variables. Abbreviations: CI, Confidence interval; ALT, alanine aminotransferase; AST, aspartate aminotransferase; CAP score, controlled attenuation parameter score; NAFLD, non-alcoholic fatty liver disease; USG, ultrasonography; LDL, low density lipoprotein; HDL, high density lipoprotein; BMI, body mass index; HOMA-IR, homeostasis model assessment technique-insulin resistance; HbA1C, glycated hemoglobin; FIB-4, fibrosis-4.

**Table 3:** Comparison of outcome values after 24 and 48 weeks between the control and MNT group.

Characteristics	Control (*n* = 21)		MNT (*n* = 27)		*p*^1^ *	*p*^2^ *
	24 w	48 w	24 w	48 w		
**Primary outcome**						
ALT (IU/L)	61 ± 25	66 ± 37	77 ± 57	57 ± 36	0.246	0.400
**Steatosis factors**						
AST (IU/L)	35 ± 11	37 ± 17	48 ± 33	36 ± 15	0.075	0.776
CAP score (dB/m)	299 ± 32	307 ± 43	297 ± 34	301 ± 44	0.831	0.642
NAFLD fat score	3.0 ± 2.5	2.9 ± 3.2	2.8 ± 2.0	2.4 ± 1.8	0.677	0.489
Hepatic steatosis index	44.5 ± 4.4	44.5 ± 4.9	43.3 ± 4.8	43.0 ± 6.1	0.389	0.354
USG fatty liver grade (%)					NA	0.574
Grade 0	NA	-	NA	1 (3.7)		
Grade I	NA	6 (28.5)	NA	11 (40.7)		
Grade II	NA	10 (47.6)	NA	7 (25.9)		
Grade III	NA	5 (23.8)	NA	8 (29.6)		
**Metabolic factors**						
Total cholesterol (mg/dL)	156 ± 43	163 ± 48	135 ± 28	152 ± 42	0.051	0.392
LDL cholesterol (mg/dL)	87 ± 31	95 ± 36	75 ± 24	87 ± 37	0.140	0.456
HDL cholesterol (mg/dL)	47 ± 9	47 ± 9	45 ± 7	48 ± 9	0.408	0.767
Triglyceride (mg/dL)	173 ± 128	166 ± 106	137 ± 47	145 ± 51	0.230	0.381
BMI (kg/m^2^)	29.8 ± 3.9	29.8 ± 3.7	29.5 ± 4.1	29.7 ± 4.5	0.845	0.974
Muscle mass (kg)	29.4 ± 6.5	29.5 ± 6.3	30.9 ± 7.1	31.1 ± 7.4	0.443	0.427
Body fat percentage (%)	35.9 ± 8.2	36.0 ± 7.2	33.5 ± 7.7	33.4 ± 8.3	0.312	0.261
HOMA-IR	121 ± 77	115 ± 99	101 ± 50	100 ± 56	0.285	0.529
Fasting glucose (mg/dL)	105 ± 15	105 ± 13	109 ± 18	108 ± 11	0.403	0.397
HbA1C (%)	NA	5.9 ± 0.4	NA	5.8 ± 0.4	NA	0.527
Serum Insulin (uIU/mL)	NA	24 ± 20	NA	20 ± 11	NA	0.442
C-peptide (ng/mL)	NA	3.4 ± 1.1	NA	3.1 ± 1.0	NA	0.365
**Fibrosis factors**						
NAFLD fibrosis score	−3.6 ± 1.3	−3.7 ± 1.5	−2.6 ± 1.1	−2.7 ± 1.3	0.012	0.017
Fibrosis score (kPa)	6.0 ± 2.4	5.5 ± 1.8	5.7 ± 2.2	5.4 ± 1.4	0.831	0.810
FIB-4	0.7 ± 0.3	0.7 ± 0.4	1.0 ± 0.5	0.8 ± 0.5	0.039	0.345

Data are presented as mean ± standard deviation in continuous variable or number (percent) in categorial variables. Proportions are presented as percentages for categorical variables. *p*-values were calculated by the Kruskal–Wallis test for continuous variables and the chi-square test or Fisher’s exact test or the Cochran–Mantel–Haenszel test for categorical variables. * *p*^1^ refers to the *p*-value calculated between control group and education group with 24-week results, and *p*^2^ refers to 48-week results. Abbreviations: ALT, alanine aminotransferase; AST, aspartate aminotransferase; CAP score, controlled attenuation parameter score; NAFLD, non-alcoholic fatty liver disease; USG, ultrasonography; NA, non-available; LDL, low density lipoprotein; HDL, high density lipoprotein; BMI, body mass index; HOMA-IR, homeostasis model assessment technique-insulin resistance; HbA1C, glycated hemoglobin; FIB-4, fibrosis-4.

## Supplementary Materials

The following are available online at https://www.mdpi.com/article/10.3390/nu13124453/s1, Figure S1: Nutrition education brochure given to control group, Table S1: Clinical Trial Visits and Clinical Trial Process Planning.
