# Supplementary material for: Effect of Nutrition Education in NAFLD Patients Undergoing Simultaneous Hyperlipidemia Pharmacotherapy: A Randomized Controlled Trial
Source: Nutrients. 2021 Dec 13;13(12):4453. doi: 10.3390/nu13124453 (PMC8709046; doi:10.3390/nu13124453)
Supplement: Supplementary file 1 [file nutrients-13-04453-s001.zip › nutrients-1499100-supplementary.pdf]

Supplementary Table 1. Clinical Trial Visits and Clinical Trial Process Planning

Procedure	Screening	Baseline/ Day 1 [1]	Treatment Period			
			Week			
			12	24	36	48
Informed consent	x					
Medical history	x					
Eligibility screen	x					
Serum HBV, HCV, HIV-1	x					
Anthropometric measures: height and weight[1]	x	x	x	x	x	x
Bioelectric impedance analysis [2]	x	x	x	x	x	x
Vital signs[3]	x	x	x	x	x	x
Urine β-HCG [4]	x					x
Routine biochemistry laboratory test[5, 6]	x	x	x	x	x	x
Ultrasonography	x					x
Transient elastography	x	x		x		x
Adverse events		x	x	x	x	x
Concomitant medication usage	x	x	x	x	x	x
Administration of antihyperlipidemic agents		x	x	x	x	
Medical nutritional therapy sessions		x	x	x	x	x

Abbreviations: HBV, hepatitis B virus; HCV, hepatitis C virus; HIV, human immunodeficiency virus; HCG, human chorionic gonadotropin

1. Height was measured only on screening day
2. Measured by Inbody 970®
3. Blood pressure, heart rate, Body temperature
4. Tested in females of childbearing age
5. Routine biochemistry laboratory test include
- Hematology: red blood cell count, white blood cell count, hemoglobin, hematocrit, platelet, neutrophil
- Biochemistry: aspartate transaminase, alanine transferase, total bilirubin, alkaline phosphatase, γ-glutamyltransferase, total protein, albumin, blood urea nitrogen, creatinine, C-reactive protein, glycated hemoglobin, fasting glucose, fasting insulin, ferritin
- Hemostasis: Prothrmobin time, activated partial thromboplastin time
- Lipid panel: Total cholesterol, low density lipoprotein, high density lipoprotein, triglyceride
- Urine analysis with microscopy (only at screening)

6. If the first administration of medication is within 2 weeks after screening, results at screening day was acknowledged as baseline results.

Participants are allowed to visit the clinic ± 14 days within planned date of visit.
However, the next visiting date is calculated from the planned visit schedule.

Supplementary Figure 1. Nutrition education brochure given to control group

지방간, 어떻게 관리해야 하나요?
-식이 및 운동 요법

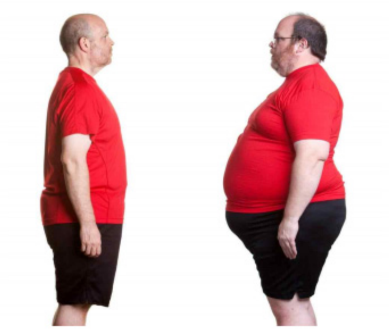

순천향대학교 부천병원
간클리닉

Q. 지방간이란?

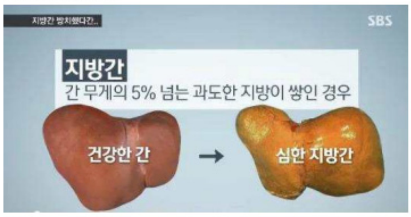

정상적인 간에는 지방이 5% 정도 존재하는데 간의 무게의 5% 이상으로 지방이 침착된 경우를 지방간이라고 합니다. 지방 중에서 중성지방(트리글리세라이드)이 간세포에 축적되는데 음식물 등을 통하여 섭취한 지방질을 원활하게 처리하지 못하여 지방간이 발생합니다.

Q. 왜 생기나요?

지방간의 4대 원인은, 과도한 음주, 비만(복부비만), 당뇨병, 고질혈증 등입니다. 지방간은 그 원인에 따라서, 알콜성 지방간과 비알콜성 지방간으로 분류됩니다. 비알코올성 지방간은 하루에 40g(4잔)이하의 음주를 하는 사람에서 지방간이 생기는 경우를 말하며, 대부분의 경우에 과체중이나 비만(복부비만), 당뇨병, 고지혈증 등의 위험요인이 있습니다. 드물게, 피임약 등 여성호르몬이나 스테로이드를 포함한 여러 가지 약제를 오래 복용하는 사람들에서 지방간을 동반하는 경우도 있습니다. 급작스런 체중감소나 체중감소를 위해 수술을 한 후에도 심한 지방간이 올 수 있습니다.

Q. 관리하지 않으면 무슨 문제가 생기죠?

지방간은 원인에 따라 다르지만 지방간염으로 진행할 수 있으며, 간경변이나 간암으로 진행하는 경우가 비교적 드물게 있을 수 있으므로 주기적 검진이 필요합니다. 계속 음주를 하거나 비만이 심해지면 간경변증으로 더 빨리 진행할 수 있습니다.

Q. 그럼 어떻게 치료할 수 있나요?

지방간의 원인을 제거하는 것이 가장 중요한 치료 방법입니다.

[술이 원인인 경우]

술이 원인일 때는 금주를 하는 것이 가장 중요합니다. 그리고 술 때문에 부족해진 영양분을 보충해 주는 것이 필요합니다. 금주와 함께 꾸준한 운동이 필요합니다. 일시적인 알코올성 지방간은 대게 문제가 안 되지만 계속 음주를 하게 되면 알코올성 간염, 간경변증으로 진행하게 됩니다. 이렇게 되면 술을 끊더라도 병의 진행을 막을 수 없게 되는 경우가 있습니다.

[비만이 원인인 경우]

• 체중감소 : 현재 체중의 10%를 3~6개월 내에 서서히 줄입니다. 너무 갑작스런 체중 감량은 오히려 지방간을 악화시킬 수 있습니다.

• 운동요법 : 운동은 지방간 치료에 도움이 될 뿐 아니라 혈압을 내리고, 혈중 콜레스테롤을 감소시키며, 혈당도 내리고, 뼈와 근육을 건강하게 해 주며 전신적인 스트레스를 해소시킵니다. 운동은 각자의 상황과 체력에 맞도록 선택하는데, 빠르게 걷기, 자전거 타기, 조깅, 수영, 등산, 에어로빅 댄스 등의 유산소운동을 꾸준히 일주일에 3번 이상, 한 번 운동 시 30분 이상 합니다. 매우 규칙적으로 운동할 수 있으면 더 좋습니다.

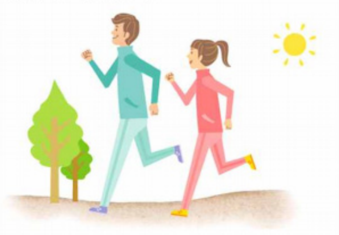

주 150분 이상 운동을 지속합니다.

• 식이요법

-기본 원칙

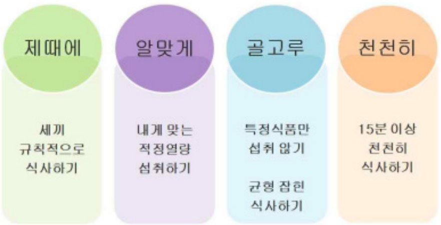

-어떻게 먹어야 할까요?

1. 과도한 당질(복합당, 단순당) 줄이고 식이섬유소의 섭취를 늘리세요.

<탄수화물 식품>-과잉 섭취시 지방으로 전환, 저장됩니다.

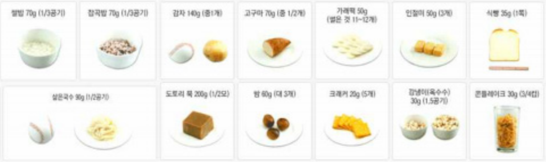

<단순당 식품>-섭취를 제한합니다.

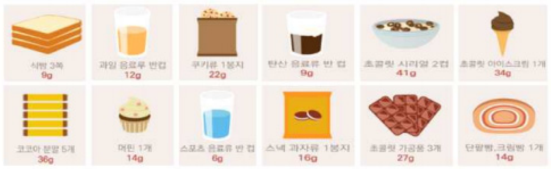

<과일류>-하루에 1/2~1주먹 내외로 섭취합니다.

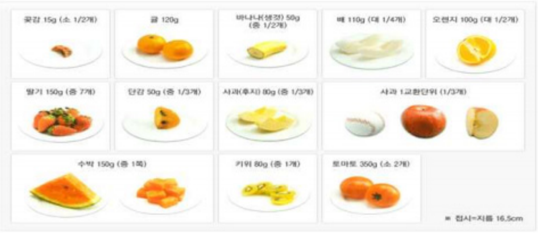

<채소류>-한끼에 2접시 이상 섭취합니다.

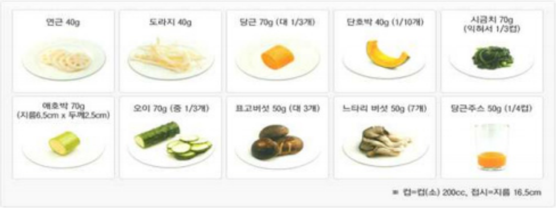

2. 지방이 간에 쌓이는 것을 예방하려면 단백질의 섭취도 중요합니다.

<단백질 식품을 한끼에 80~100g 내외로 섭취합니다.>

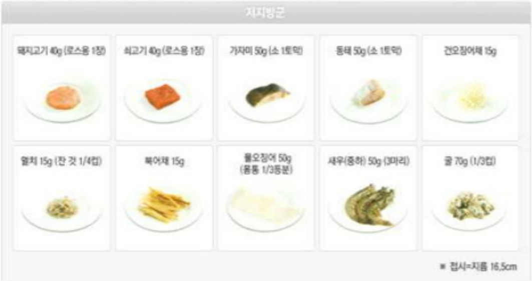

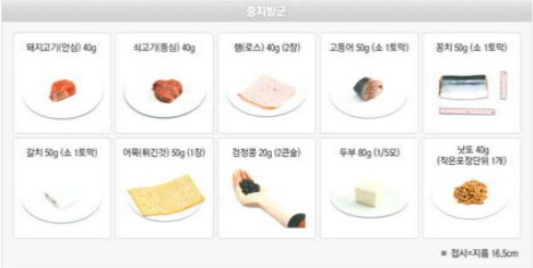

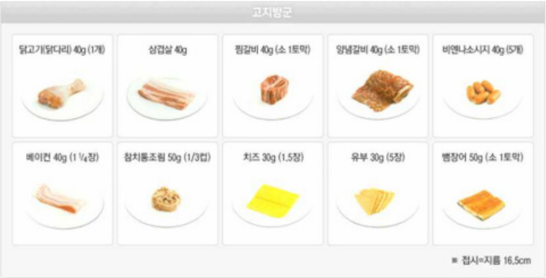

3. 지방, 총 섭취량 뿐만 아니라 종류도 신경을 써야 합니다.

<고지방 식품 및 부위를 피합니다.>

프라이드 치킨	→	껍질 벗긴 전기구이 통닭
감자튀김	→	찐 감자
볶음밥	→	비빔밥
갈비구이	→	생등심 구이(기름제거)
튀김가락국수	→	메밀 국수
생선커틀렛	→	생선구이
팝콘	→	강냉이
자장면	→	기스면
군만두	→	물만두
아이스크림	→	셔벗
계란 프라이	→	반숙, 찜
나물볶음	→	생채, 무침

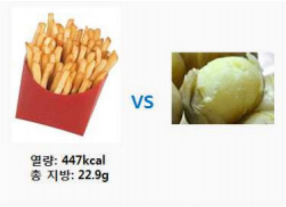

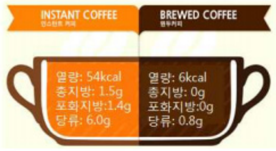

<포화지방산은 지방간의 주요한 위험요인이므로 줄이며, 불포화지방산은 충분히 섭취합니다.>

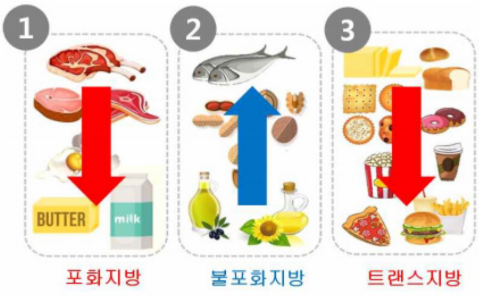

4. 콜레스테롤이 많이 함유된 식품의 섭취를 제한합니다.

<1일 콜레스테롤 섭취량은 300mg이하로 제한하고, 콜레스테롤이 많이 함유된 식품은 1주일에 1~2회 이하로 섭취합니다.>

콜레스테롤이 많은 식품(1주일에 1~2회 이하 섭취)
간, 곱창, 달걀노른자, 오징어, 새우, 장어, 미꾸라지, 생선 알과 내장

5. 염분의 섭취를 줄입니다.

<혈압 조절 및 심장 혈관에 부담을 적게하기 위해 음식을 싱겁게 섭취하며, 염분이 많은 식품의 섭취에 주의 합니다.>

염분 함량이 높은 식품
김치, 장아찌, 단무지, 젓갈, 국/찌개류, 장류(된장, 고추장, 간장)

※지방관리를 위한 하루 식단!

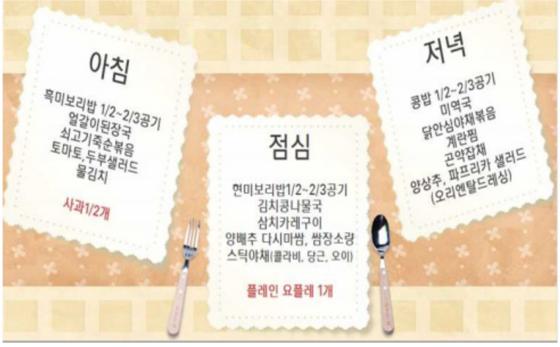

※ 똑똑한 외식메뉴 선택방법!

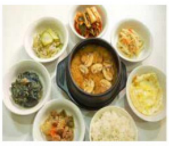
백반

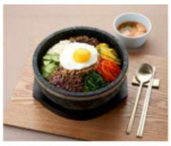
비빔밥

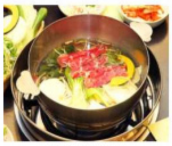
샤브샤브

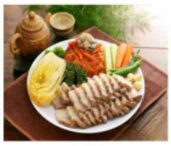
보쌈

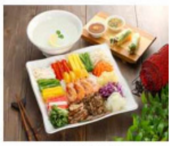
월남쌈
